# Neurological Complications of Veno-Arterial Extracorporeal Membrane Oxygenation: A Retrospective Case-Control Study

**DOI:** 10.3389/fmed.2021.698242

**Published:** 2021-07-01

**Authors:** Yinan Luo, Qiao Gu, Xin Wen, Yiwei Li, Weihua Peng, Ying Zhu, Wei Hu, Shaosong Xi

**Affiliations:** Department of Critical Care Medicine, Affiliated Hangzhou First People's Hospital, Zhejiang University School of Medicine, Hangzhou, China

**Keywords:** V-A ECMO, neurological complications, retrospective study, risk indicators, long-term outcomes

## Abstract

**Background:** To explore the epidemiology, clinical features, risk indicators, and long-term outcomes of neurological complications caused by veno-arterial extracorporeal membrane oxygenation (V-A ECMO).

**Methods:** We retrospectively analyzed 60 adult patients who underwent V-A ECMO support in our unit from February 2012 to August 2020. These patients were separated into the neurological complications group (NC group) and the non-neurological complications group (nNC group). The differences in basic data and ECMO data between the two groups were compared. The data of long-term neurological prognosis were collected by telephone follow-up.

**Results:** Thirty-nine patients (65.0%) had neurological complications. There were significant differences between the two groups in terms of median age, hypertension, median blood urea nitrogen, median troponin I (TNI), median lactic acid, pre-ECMO percutaneous coronary intervention, continuous renal replacement therapy (CRRT), median Sequential Organ Failure Assessment score, median Acute Physiology and Chronic Health Evaluation II score, median peak inspiratory pressure, median positive end expiratory pressure, and median fresh frozen plasma (*P* < 0.05). The median Intensive Care Unit length of stay (ICU LOS), 28-day mortality, median post-ECMO vasoactive inotropic score, non-pulsate perfusion (NP), and median ECMO duration of the NC group were significantly higher than those of the nNC group (*P* < 0.05). Furthermore, multiple logistic regression analysis revealed that TNI (*P* = 0.043), CRRT (*P* = 0.047), and continuous NP > 12 h (*P* = 0.043) were independent risk indicators for neurological complications in patients undergoing ECMO. Forty-four patients (73.3%) survived after discharge, and 38 patients (63.3%) had Cerebral Performance Category score of 1–2. And there were significant differences between the two groups in long-term neurological outcomes after discharge for 6 months (*P* < 0.05).

**Conclusion:** The incidence of neurological complications was higher in patients undergoing V-A ECMO and was closely related to adverse outcomes (including ICU LOS and 28-day mortality). TNI, CRRT, and continuous NP > 12 h were independent risk indicators for predicting neurological complications in ECMO supporting patients. And the neurological complications of patients during ECMO support had significant adverse effect on long-term surviving and neurological outcomes of patients after discharge for 6 months.

## Introduction

Veno-arterial extracorporeal membrane oxygenation (V-A ECMO) can replace the roles of the heart and lungs to maintain circulation and respiration and is used to treat acute cardiac or pulmonary failure. However, ECMO support can cause various complications due to the severity of the diseases and longstanding extracorporeal circulation, which may negatively impact patients' survival (1, 2).

The Extracorporeal Life Support Organization (ELSO) registry reported that survival after ECMO had reached to 58% from 1989 (3). Mortality and poor functional outcomes are often induced by neurological injury that results not only from underlying diseases but also from complications associated with ECMO support itself (4, 5). With ECMO being widely used, multiple studies on ECMO have focused on neurological complications, including cognitive dysfunction, hypoxic-ischemic encephalopathy, and even cerebral ischemic stroke and cerebral hemorrhage (6–8). However, a large knowledge gap exists in our understanding and treatment of ECMO-related neurological complications. The data about epidemiology, pathophysiology, and risk indicators of neurological complications is limited, meanwhile, no practice guidelines or management strategies for the neurological care of ECMO patients (9, 10).

Through retrospectively analyzing patients with V-A ECMO support in our unit, we aimed to investigate the epidemiology, clinical features, and risk indicators of neurological complications caused by V-A ECMO supporting.

## Methods

### Study Design and Participants

We collected data on all in-hospital and out-of-hospital adult (>18 years old) patients who received V-A ECMO support at the Department of Critical Care Medicine, Affiliated Hangzhou First People's Hospital, Zhejiang University School of Medicine, from February 2012 to August 2020. The inclusion and exclusion criteria were determined based upon current reports and the clinical experience of our unit.

Inclusion criteria:

(1) Time nodes: Process of ECMO support, and after weaning from ECMO.
(2) Types of neurologic complications: Short-term or persistent mental and organic diseases observed after stopping sedative for 48 h, including coma, delirium, depression, epilepsy, hypoxic ischemic encephalopathy, ischemic stroke, hemorrhagic stroke and death, that were identified by the Glasgow Coma Scale (GCS <15, patients with endotracheal intubation <11), the Cerebral Performance Category (CPC score > 2), the confusion assessment method for the ICU (CAM-ICU), and the neuroimaging examination.

Exclusion criteria

(1) Acute primary craniocerebral injury before admission, or previous neuropsychic symptoms.
(2) Incomplete and missing cases.
(3) Duration of ECMO support <24 h.

### Data Collection

(1) Baseline characteristics: Age; sex; underlying diseases, including hypertension, diabetes, and coronary heart disease (CHD); etiology supporting the use of ECMO; hemodynamic data such as mean arterial pressure (MAP) and Central Venous Pressure (CVP); biochemical indexes (blood gas analysis, blood biochemistry, coagulation function, blood routine) 24 h post-ECMO support; assessment of severity after ECMO support for 24 h, including Acute Physiology and Chronic Health Evaluation II (APACHE-II) score, Sequential Organ Failure Assessment (SOFA) score; and other interventions, including percutaneous coronary intervention (PCI), intra-aortic balloon pump (IABP), mechanical ventilation (MV), and continuous renal replacement therapy (CRRT).
(2) ECMO-related characteristics: Location of ECMO, duration of building ECMO, vasoactive inotropic score [VIS = dose of dopamine + dose of dobutamine + 100 × dose of epinephrine + 10 × dose of milrinone + 10,000 × dose of vasopressin + 100 × dose of norepinephrine (unit: μg/kg/min)], duration of non-pulsatile perfusion (NP) after ECMO support, ECMO duration, mechanical ventilation parameters, complications, dosage of blood product [red blood cell (RBC) and fresh frozen plasma (FFP)], and weaning from ECMO
(3) Outcome indicators: Intensive Care Unit (ICU) length of stay (LOS), hospital LOS, 28-day mortality, incidence of neuropathy and mortality after discharge.
(4) Data processing: The epidemiology, clinical features, and related risk indicators connected with the identified neurological complications are discussed through the analysis of the above data.

### Extracorporeal Life Support Technology

All patients used the ROTAFLOW centrifugal pump and piping system produced by MAQUET, Germany, and all modes of connection were V-A ECMO. Furthermore, all patients received a peripherally inserted catheter into the femoral artery and femoral vein under ultrasound guidance. The left heart function of ECMO-assisted patients was evaluated via cardiac ultrasound and the circulatory state. An IABP was implemented when necessary. The application of CRRT depended on renal function, urine volume, and intake and output volume management. All patients received tracheal intubation and mechanical ventilation, and periodical and individualized analgesics-sedatives.

### Statistical Analysis

All data were statistically processed using SPSS 25.0 statistical software. Categorical variables and continuous variables are represented as counts (%) and medians [inter quartile range (IQR)]. Chi-square or Fisher's exact test was used for categorical variables, and the student's *t*-test or Mann–Whitney *U* test was used for continuous variables. Multiple logistic regression analysis was used to analyze statistically significant variables to identify independent risk indicators related to neurological complications, which are summarized as odds ratios (OR) and 95% confidence intervals (95%CI). *P*-values < 0.05 were considered statistically significant.

## Results

### Comparison of Baseline Characteristics of Patients

**Figure 1:**
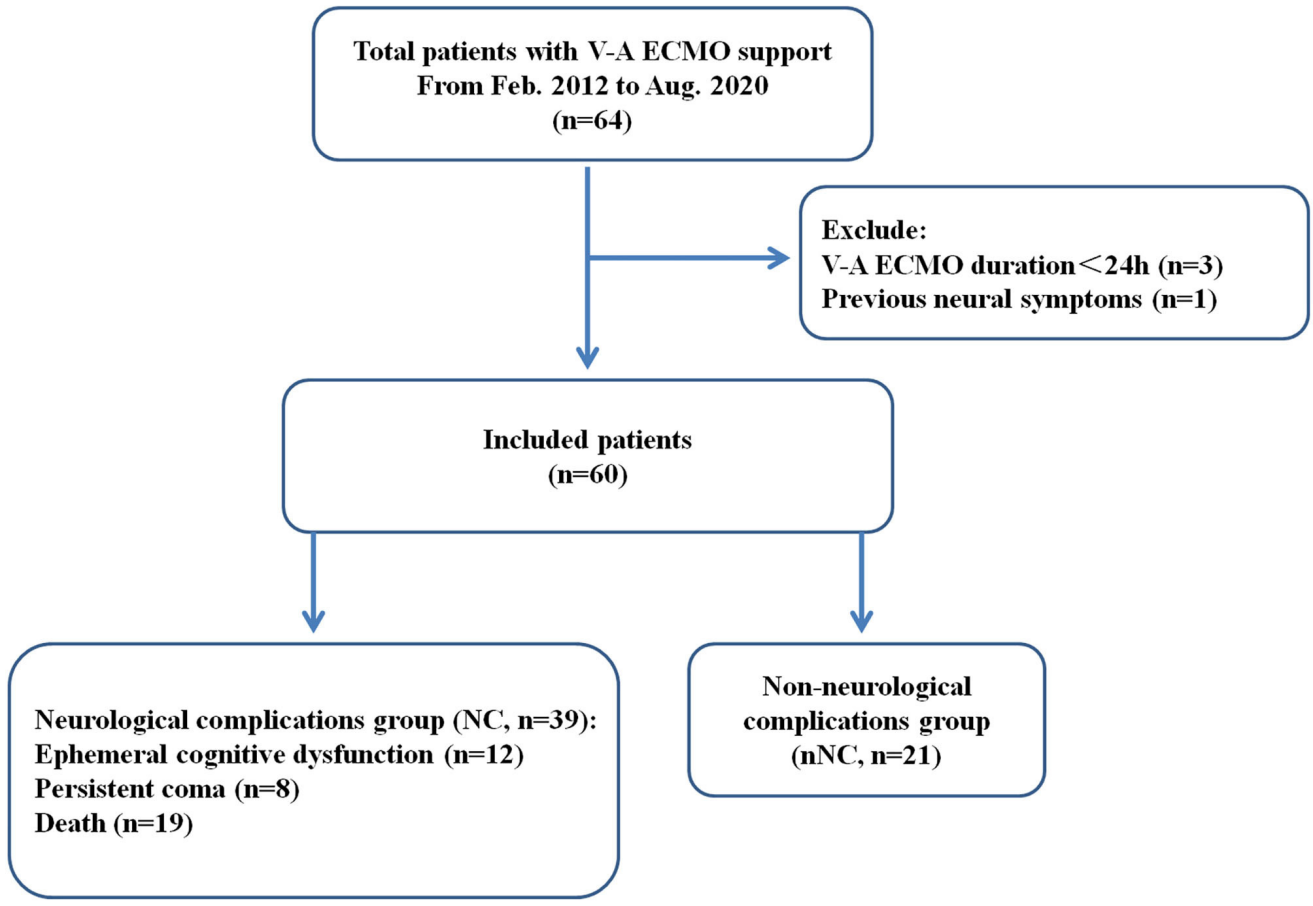
Flow chart of patient cohorts. V-A ECMO, veno-arterial extracorporeal membrane oxygenation; NC, neurological complications group; nNC, non-neurological complications group.

After excluding four patients, a total of 60 patients with V-A ECMO support were retrospectively screened and assigned to the neurological complications group (NC group) and the non-neurological complications group (nNC group) based on the presence or absence of neurological complications (Figure 1). Of these 60 patients, 39 patients (65.0%) suffered neurological complications, including ephemeral cognitive dysfunction (*n* = 12, including brief coma and delirium), persistent coma (*n* = 8, hypoxic-ischemic encephalopathy) and death (*n* = 19) (Table 1). The median ages of the patients in the NC and nNC groups were 50 [31, 66] and 30 [24, 35] years old, respectively, with the NC group patients being significantly older than the nNC group patients (*P* < 0.01). But there was not significant statistical difference between the two groups in some pre-ECMO baseline characteristics (Supplementary Table S2). In addition, patients were considered to be more likely to develop neurological complications when their etiologies for ECMO support were acute myocardial infarction (*P* < 0.001) and acute fulminant myocarditis (*P* < 0.001), and when the underlying diseases were hypertension (*P* = 0.011) and diabetes (*P* = 0.042). We also found significant differences 24 h post-ECMO support between the NC and nNC groups with respect to the median concentration of blood urea nitrogen (BUN) (8.12 [605, 13.81] vs. 6.43 [4.45, 8.71] mmol/L, *P* = 0.012), median concentration of troponin I (TNI) (16.7 [1.7, 88.8] vs. 5.0 [1.5, 7.5] μg/L, *P* = 0.041), median concentration of lactic acid (LAC) (1.8 [1.3, 3.3] vs. 1.4 [1.0, 2.0] mmol/L, P = 0.03), SOFA score (12 [10, 14] vs. 9 [5, 10], P < 0.001), and APACHE-II score (22 [17, 27] vs. 13 [7, 18], *P* < 0.001). Meanwhile, the results showed the proportion of patients who underwent PCI after ECMO support (28.2 vs. 4.8%, *P* = 0.042), and the CRRT during ECMO support (69.2 vs. 28.6%, *P* = 0.003) was higher in the NC group than in the nNC group (Table 1). Besides, we investigated the ECMO flow, MAP, CVP and blood gas of patients during the phase of pre- and post- ECMO support, and the results did not show significant statistical difference between the groups with and without neurological complications (*P* > 0.05, Table 2 and Supplementary Tables S1–2).

**Table 1:** Comparison of baseline characteristics of patients.

	**All patients**	**Neurological complications**		***P***
	***n* = 60**	**Yes (*n* = 39)**	**No (*n* = 21)**	
**Age (years)**	39 (29, 59.5)	50 (31, 66)	30 (24, 35)	<0.001
**Sex**, ***n*** **(%)**				
Male	36 (60)	26 (66.7)	10 (47.6)	0.176
Female	24 (40)	13 (33.3)	11 (53.4)	
**Underlying diseases**, ***n*** **(%)**				
Hypertension	15 (25)	14 (35.9)	1 (4.8)	0.011
Diabetes	8 (13.3)	8 (20.5)	0 (0)	0.042
CHD	5 (8.3)	5 (12.8)	0 (0)	0.152
**Initiate etiology**, ***n*** **(%)**				
AMI	22 (36.7)	21 (53.8)	1 (4.8)	<0.001
AFM	29 (48.3)	12 (30.8)	17 (81.0)	<0.001
MA	3 (5.0)	2 (5.1)	1 (4.8)	1
CA	29 (48.3)	22 (56.4)	7 (33.3)	0.109
Others	6 (10.0)	4 (10.3)	2 (9.5)	1
**24 h post-ECMO**				
MAP (mmHg)	79 (72.1, 87.9)	77.7 (71.7, 85.3)	82.3 (73.8, 91.5)	0.059
CVP (cmH_2_O)	9 (6, 12)	9.5 (6, 13)	7 (5, 12)	0.371
PH	7.448 (7.409, 7.49)	7.456 (7.409, 7.488)	7.44 (7.408, 7.501)	0.567
PaO_2_ (mmHg)	200 (131, 302.9)	193 (129.1, 390.3)	233 (136, 296.1)	0.620
PaCO_2_ (mmHg)	31.9 (27.5, 34.9)	32.2 (28.3, 35)	31 (23.8, 34)	0.205
ScvO_2_ (mmHg)	74.5 (66.9, 82.9)	70.9 (66.4, 80.3)	81 (71, 84.7)	0.091
BUN (mmol/L)	7.26 (5.74, 10.14)	8.12 (6.05, 13.81)	6.43 (4.45, 8.71)	0.012
K (mmol/L)	4.31 (4.05, 4.53)	4.32 (4.11, 4.59)	4.29 (4.02, 4.46)	0.426
Cr (μmol/L)	105.5 (84.5, 134)	108 (94, 156)	102 (75, 117.5)	0.083
TBil (μmol/L)	20.1 (13.5, 37.4)	23 (15.1, 37.5)	15.4 (11.2, 26.5)	0.104
GLU (mmol/L)	9.15 (7.32, 10.91)	9.26 (6.69, 10.52)	8.81 (8.38, 11.41)	0.248
Hb (g/L)	94 (77, 117.25)	94 (75, 115)	94 (78.5, 119.5)	0.587
PT (s)	16.3 (14.1, 20.5)	17 (14.3, 21.7)	15.4 (14, 19.7)	0.327
APTT (s)	93.2 (60.8, 141.7)	93.2 (58.5, 134.1)	96.7 (62.3, 157.3)	0.934
TNI (μg/L)	6.71 (1.6, 45.4)	16.7 (1.7, 88.8)	5 (1.5, 7.5)	0.041
LAC (mmol/L)	1.7 (1.2, 2.6)	1.8 (1.3, 3.3)	1.4 (1, 2)	0.03
CRP (mg/L)	61.5 (28.75, 81.75)	67 (32, 92)	52 (18.5, 70)	0.097
PCT (μg/L)	2.59 (0.27, 22.75)	4.28 (0.38, 37.4)	0.69 (0.14, 6.61)	0.052
**Pre-ECMO score**				
SOFA score	10 (7, 12)	11 (8, 12)	8 (4, 10)	0.004
APACHE- II score	19.5 (12, 25)	22 (18, 31)	12 (6, 16.5)	<0.001
**24 h post-ECMO score**				
SOFA score	11 (8, 14)	12 (10, 14)	9 (5,10)	<0.001
APACHE- II score	19 (13, 24)	22 (17, 27)	13 (7, 18)	<0.001
**Other intervenes**, ***n*** **(%)**				
Pre-ECMO PCI	7 (11.7)	7 (17.9)	0 (0.0)	0.085
Post-ECMO PCI	12 (20.0)	11 (28.2)	1 (4.8)	0.042
IABP	25 (41.7)	19 (48.7)	6 (28.6)	0.174
Pre-ECMO MV	50 (83.3)	34 (87.2)	16 (76.2)	0.298
Post-ECMO MV	10 (16.7)	5 (12.8)	5 (23.8)	0.298
CRRT	33 (0.55)	27 (69.2)	6 (28.6)	0.003
**Outcomes**				
ICU LOS (days)	14 (10, 21)	16 (11, 24)	11 (9, 17.5)	0.038
Hospital LOS (days)	19.5 (13, 27)	19 (13, 30)	20 (13, 26.5)	0.981
28-day mortality (%)	20 (33.3)	20 (51.3)	0 (0.0)	<0.001

*CHD, coronary heart disease; AMI, acute myocardial infarction; AFM, acute fulminant myocarditis; CA, cardiac arrest; ECMO, extracorporeal membrane oxygenation; MAP, mean arterial pressure; CVP, Central Venous Pressure; ScvO_2_, Central venous oxygen saturation; PH, Potential of Hydrogen; PaO_2_, Arterial oxygen partial pressure; PaCO_2_, Arterial carbon dioxide partial pressure. BUN, blood urea nitrogen; K, kalium; Cr, creatinine; TBIL, total bilirubin; GLU, glucose; Hb, hemoglobin; PT, prothrombin time; APTT, activated partial thromboplastin time; TNI, troponin I; LAC, lactic acid; CRP, C-reactive protein; PCT, procalcitonin; SOFA, sequential organ failure assessment; APACHE- II, acute physiology and chronic health evaluation II; PCI, percutaneous coronary intervention; IABP, intra-aortic balloon pump; MV, mechanical ventilation; CRRT, continuous renal replacement therapy; LOS, length of stay*.

**Table 2:** Comparison of V-A ECMO related characteristics.

	**All patients**	**Neurological complications**		***P***
	***n*=60**	**Yes (*n*=39)**	**No (*n*=21)**	
**ECPR**, ***n*** **(%)**	29 (48.3)	22 (56.4)	7 (33.3)	0.109
**Locations of ECMO**, ***n*** **(%)**				
OR	7 (11.7)	6 (15.4)	1 (4.8)	0.404
ICU	49 (81.7)	31 (79.5)	18 (85.7)	0.078
ED	4 (6.7)	2 (5.1)	2 (9.5)	0.287
**Duration of building ECMO (mins)**	53.5 (40, 67.75)	51 (40, 61)	55 (40, 71.5)	0.571
**ECMO flow (L/min)**				
Initial Flow	3.63 (3.21, 4.18)	3.72 (3.33, 4.15)	3.49 (3.05, 4.35)	0.803
24 h post-ECMO	3.34 (3.00, 3.97)	3.40 (3.00, 4.01)	3.55 (3.00, 3.95)	0.845
48 h post-ECMO	3.48 (3.06, 3.97)	3.44 (3.09, 4.11)	3.55 (2.98, 3.95)	0.607
**VIS, mean**				
0 h post-ECMO	36 (8.5, 123)	60 (20, 181.1)	15 (4, 57)	0.013
24 h post-ECMO	10 (0, 19.2)	12 (3, 20.8)	3.8 (0, 17.2)	0.094
**Continuous NP** **>** **12 h**, ***n*** **(%)**	15 (25.0)	14 (35.9)	1 (4.8)	0.011
**ECMO duration (days)**	6 (5, 8)	7 (5, 11)	5 (4, 6)	0.01
**MV parameter at 24 h post-ECMO**				
FiO_2_ (%)	100 (62.5, 100)	90 (70, 100)	100 (50, 100)	0.874
RR (times/min)	12 (12, 16)	12 (12, 15)	12 (12, 17)	0.672
PIP (cmH_2_O)	20 (16, 22)	21 (18, 24)	16 (15, 20)	0.003
PEEP (cmH_2_O)	8 (7, 10)	10 (8, 10)	7 (5, 8)	0.004
**Complication**, ***n*** **(%)**				
Cannulation site bleeding	42 (70.0)	28 (71.8)	14 (66.7)	0.771
Limb ischemia	6 (10.0)	5 (12.8)	1 (4.8)	0.412
**Dosage of blood products**				
RBC (U)	4.0 (2.0, 7.0)	4 (2, 6)	3 (0, 9.25)	0.33
FFP (ml)	520 (205, 1035)	660 (250, 1150)	340 (0,925)	0.043
**Successful weaning from ECMO**, ***n*** **(%)**	50 (83.3)	29 (74.4)	21 (100.0)	0.011

*ECPR, extracorporeal cardiopulmonary resuscitation; ECMO, extracorporeal membrane oxygenation; OR, operation room; ED, emergency department; VIS, vasoactive inotropic score [= dose of dopamine + dose of dobutamine + 100 × dose of epinephrine + 10 × dose of milrinone + 10,000 × dose of vasopressin + 100 × dose of norepinephrine (unit: μg/kg/min)]; NP, non-pulsate perfusion; MV, RR: respiratory rate; PIP, peak inspiratory pressure; PEEP, positive end expiratory pressure; RBC, red blood cell; FFP, fresh frozen plasma*.

The primary adverse outcome were ICU LOS, hospital LOS, and 28-day mortality. The results showed no significant differences in hospital LOS between the NC and nNC groups. However, we found that the ICU LOS (16 [11, 24] vs. 11 [9, 17.5] days, *P* = 0.038) and the 28-day mortality (51.3% vs. 0, *P* < 0.001) of the NC group were significantly higher than those of the nNC group (Table 1).

### Comparison of V-A ECMO-Related Characteristics

As shown in Table 2, 29 patients (48.3%) underwent extracorporeal cardiopulmonary resuscitation (ECPR), which was not significantly related to the development of neurological complications. The locations of ECMO surgery included the operation room (*n* = 7, 11.7%), ICU (*n* = 49, 81.7%), and emergency department (*n* = 4, 6.7%); the location was not significantly related to the development of neurological complications. However, we found significant differences between the NC and nNC groups with respect to the median VIS (60 [20, 181.1] vs. 15 [4, 57], *P* = 0.013) at the 0h post-ECMO, the median ECMO duration (7 [5, 11] vs. 5 [4, 6] days, *P* = 0.01), and the median FFP dosage (660 [250, 1150] vs. 340 [0, 925] mL, *P* = 0.043) (Table 2).

It is worth noting that 15 patients (25.0%) suffered from non-pulsatile perfusion (NP; pulse pressure <10 mmHg) for more than 12 h after ECMO support, of whom, 14 patients suffered neurological complications, which was significantly higher than control group (35.9 vs. 4.8%, *P* = 0.011). We also investigated the mechanical ventilation parameters 24 h post-ECMO, and the results showed that the median peak inspiratory pressure (PIP) (21 [18, 24] vs. 16 [15, 20] cmH_2_O, *P* = 0.003) and positive end expiratory pressure (PEEP) (10 [8, 10] vs. 7 [5, 8] cmH_2_O, P = 0.004) in the NC group were significantly higher than those in the nNC group (Table 2).

### Multivariate Analysis of Neurological Complications in V-A ECMO Patients

Based on the above bivariate analysis in Table 1 and Table 2, the association were checked in the multivariable model, and after adjustment for age, initiate etiology, SOFA score, and APACHE- II score, the multivariable analysis revealed that the 24 h post-ECMO TNI value (OR, 1.038; 95% CI, 1.001–1.076; *P* = 0.043), CRRT (OR, 3.884; 95% CI, 1.018–14.812; *P* = 0.047), and continuous NP > 12 h (OR, 10.127; 95% CI, 1.073–95.564; *P* = 0.043) were independent risk indicators for predicting the occurrence of neurological complications in V-A ECMO patients (Table 3).

**Table 3:** Multivariate analysis of neurological complications in V-A ECMO patients.

	**OR**	**95% CI**	***P***
TNI	1.038	1.001–1.076	0.043
CRRT	3.884	1.018–14.812	0.047
Continuous NP > 12 h	10.127	1.073–95.564	0.043

*TNI, troponin I; CRRT, continuous renal replacement therapy; NP, non-pulsate perfusion*.

### Long-Term Follow-Up Outcomes of Survivors After Discharge

As shown in Table 4, 44 patients (73.3%) survived 1 month after discharge, with 6 (13.6%) patients had significant neurological damage (CPC score of 3–5). And 4 patients (9.1%) died within one month after discharge because of severe hypoxic ischemic encephalopathy (HIE) or abandoning maintenance treatment. After discharge for 3 and 6 months, 40 (66.7%) and 39 (65%) patients were surviving, with 5.0 and 2.6% of them had significant neurological damage (CPC score of 3–5), respectively. Besides, the main neurological complications at 3 months and 6 months after discharge were Hypomnesia, accounting for 12.5 and 10.3%, respectively. Other neurological complications included HIE, stroke, and peripheral neuropathy (PN) and so on.

**Table 4:** Long-term neurological outcomes of survivors after discharge.

	**Time after discharge**		
	**1 month (*n* = 44)**	**3 months (*n* = 40)**	**6 months (*n* = 39)**
**CPC score**, ***n*** **(%)**			
CPC 1–2	38 (86.4)	38 (95.0)	38 (97.4)
CPC 3–5	6 (13.6)	2 (5.0)	1 (2.6)
**Neuropathy**, ***n*** **(%)**			
HIE	1 (2.3)	1 (2.5)	1 (2.6)
Stroke	1 (2.3)	1 (2.5)	1 (2.6)
Hypomnesia	5 (11.4)	5 (12.5)	4 (10.3)
PN	1 (2.3)	1 (2.5)	1 (2.6)
Others	2 (4.5)	1 (2.5)	1 (2.6)
Death, n (%)	4 (9.1)	1 (2.5)	0 (0.0)

*CPC, Cerebral Performance Category; HIE, hypoxic ischemic encephalopathy; PN, peripheral neuropathy*.

**Figure 2:**
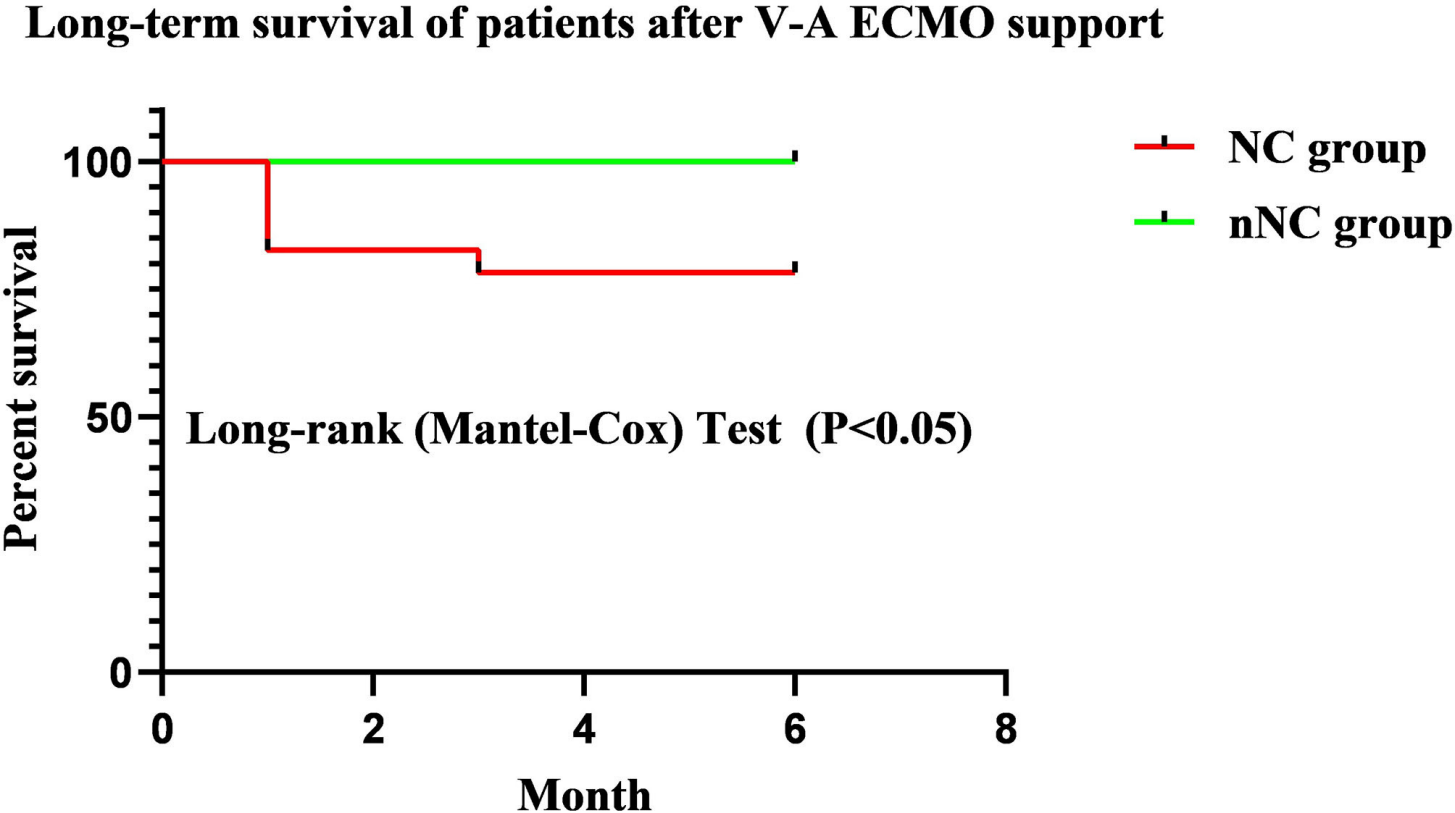
Kaplan–Meier survival analysis of patients after discharge for 6 months. The NC group had a significantly poorer 6-month survival than nNC group (HR = 7.900, 95% CI: 1.298~48.08; *P* < 0.05). NC, neurological complications; nNC, non-neurological complications group.

A Kaplan–Meier survival analysis further confirmed that the NC group had a significantly poorer 6-month survival than nNC group (HR = 7.900, 95%CI: 1.298~48.08; *P* < 0.05, Figure 2). And the neurological complications of patients during ECMO support had significant adverse effect on long-term neurological outcomes of patients after discharge for 6 months (*P* < 0.05, Table 5).

**Table 5:** Effect of neurological complications on long-term neurological outcomes.

**Neurological complications**	**Long-term outcomes after discharge**		
	**1 month (*n* = 44)**	**3 months (*n* = 44)**	**6 months (*n* = 44)**
**Yes**, ***n*** **(%)**	11 (25.0)	11 (25.0)	10 (22.7)
**No**, ***n*** **(%)**	3 (6.8)	3 (6.8)	3 (6.8)
*P*	0.024	0.024	0.049

## Discussion

V-A ECMO is a promising rescue therapy for patients with cardiac shock, with or without respiratory failure. Researchers have focused on the neurological complications and adverse outcomes in V-A ECMO supported patients (2, 11, 12). In the present study, we not only evaluated long-term neurological outcomes but also neurological organic lesions and transient psychiatric symptoms during ECMO supporting or within 28 days after. A CPC score of 1–2 was regarded as a good neurological outcome in several of these studies (11, 13). GCS and CAM-ICU were mainly used to recognize short-term neurological complications during the ECMO support. Besides, the transfer of patients with ECMO support between departments inevitably involves high-risk or immediate-threat-of-life situations that have to be dealt with immediately, sometimes within seconds (14). Therefore, the patients were not transferred to the imaging department unless there was a clear indication. In view of this, it is necessary to increase the use of bedside objective indicators, such as craniocerebral ultrasound and bedside Video-electroencephalogram (VEEG), to allow neurological complications to be easily recognized in future. These techniques can identify neurological organic lesions and psychiatric symptoms over time through calculating cerebral blood flow velocity (CBFV) and monitoring brain electrical activity. We will investigate the value of point of care ultrasound (PoCUS) combined with multimodal brain monitoring guided ECMO management in improving the outcomes of patients in future study. Moreover, biomarkers of brain injury (like NSE and S-100β) contribute to the assessment of central nervous system injury (15–17).

Sadhwani et al. described early neurodevelopmental outcomes in children who received ECMO support for cardiac indications, and demonstrated that these patients had significant developmental delays (18). In the present study, 65% patients had neurological complications. Meanwhile, the 28-day mortality of the NC group reached up to 51.3% and was significantly higher than that of the nNC group. We also found a significant difference in ICU LOS but no significant difference in hospital LOS, which further suggested that neurological complications caused by ECMO might impact mid-long-term prognosis and life quality. A recent systematic review and meta-analysis involving 6261 ECPR patients showed that the overall survival rate after ECPR was 29%, with good neurologic outcome achieved in 24% (12). However, as for the ECPR in our study, there was no significant difference between the two groups, which may be due to the small sample size.

Serum TNI is often used for estimating myocardial injury, and serious damage can result in low cardiac output (CO). A previous study of children with myocarditis showed that abnormal TN in the first 72 h of hospitalization was associated with the use of ECMO (19). Another small retrospective study evaluated 34 patients with post cardiotomy ECMO for low CO and found that a plateau in TNI levels at 48 h appeared to indicate a poor outcome due to irreversible myocardial damage (20). In the present study, the median TNI level of the NC group (16.7) was distinctly higher than that of the nNC group (5.0), and we also found there were 12 AMI patients had to undergo PCI with ECMO support because of refractory cardiogenic shock (Table 1), of which 11 patients suffered neurological complications (*P* < 0.05). An elevated TNI level in V-A ECMO patients signifies cardiac injury, which results in a drop in CO (20, 21). This reduction in CO causes lowered cerebral blood flow (CBF) and subsequent neurological complications (22).

It has been confirmed that the combination of V-A ECMO and CRRT is feasible and appears to be a safe and effective technique that has the potential to improve the fluid balance and electrolyte disturbances (23–25). A single-center retrospective chart review had found that the mortality rate of patients with combined ECMO and CRRT was higher than that of those receiving ECMO alone (26). A number of studies have indicated that damaged kidneys could have a detrimental effect on the central nervous system in acute kidney injury (AKI), which was also found to be a risk factor for delirium and coma during critical illness (27–29). In the present study, the proportion of CRRT in the NC group (69.2%) was significantly higher than that in the nNC group (28.6%), and CRRT was one of the independent risk indicators for V-A ECMO patients with neurological complications. In the meantime, we also found that the Cr of NC group was significantly higher than that of nNC group at 12, 48 and 72h after ECMO support (Supplementary Table S3). Previous study has shown neurological complications exist in the majority of patients with renal failure, and many of their effects are more obvious when renal failure acute attack (30). Epidemiological studies also exhibited an association between AKI and a subsequent risk for developing stroke and dementia (29). Especially, the dialysis-requiring AKI was associated with a higher risk and higher severity of subsequent stroke events and dementia (31, 32).

It has been reported that V-A ECMO might damage the autoregulation of CBF and result in neurological dysfunction (33, 34). We found that 25.0% patients had a duration of NP of > 12 h; NP was defined as a pulsatile pressure <10 mmHg during V-A ECMO support, referring to the paper of Yang et al. (35). Blood pressure management is crucial for patients undergoing V-A ECMO. A previous retrospective study evaluated the MAP of 116 patients receiving V-A ECMO, and the results showed that the survival of patients on V-A ECMO was significantly greater, with a higher MAP and without being affected by prolonged vasopressor use (36). Previously, Park et al. and Pappalardo et al. indicated that a higher MAP was an independent predictive factor for survival and successful weaning from V-A ECMO (37, 38). Several studies have confirmed that V-A ECMO combined with IABP could improve outcomes, enhance survival, and facilitate weaning from V-A ECMO during cardiogenic shock and cardiac arrest (39–42). Furthermore, the use of IABP could decrease the CBF with cardiac stun, and increase CBF without cardiac stun during V-A ECMO support (35). Therefore, the abovementioned studies suggested that continuous NP and low CBF might play an important role in the occurrence of neurological complications during V-A ECMO support.

Published data have exhibited persistent functional deficits associated with ECMO support (43–45). The long-term neurological sequelae of patients after weaning from V-A ECMO included hypoxic-ischemic brain injury, ischemic stroke, intracranial hemorrhage, posterior reversible encephalopathy syndrome, intracranial hypertension, seizures and brain death (10). With the overall increase in the use of ECMO, improving outcomes will depend on precisely defining the extent and types of neurologic complications and underlying pathophysiology that are specific to ECMO (10). Currently, we cannot address their etiologies with specific, targeted monitoring techniques and interventions. Furthermore, long-term survival in patients receiving ECMO was acceptable (46–48).

## Limitations

This retrospective case-control study obtained some meaningful results for clinical guidance. However, there remain some limitations. Firstly, it is a single-center retrospective study, with small sample size and lower freedom degree, which might cause statistical bias. In addition, we should add more objective indicators, including PoCUS combined with multimodal brain monitoring and cerebral regional tissue oxygenation (rSO2) monitoring guided ECMO management, to facilitate more accurate and timely recognition of neurological complications. Moreover, the absence of stratification analysis about the influence of pulsate bold flow and MAP on brain perfusion, might also cause statistical bias. Therefore, strict randomized clinical trials or substitutive research designs are necessary to reduce bias further and then clarify the neurological complications caused by V-A ECMO.

## Conclusion

In this retrospective case-control study, we found that the morbidity of neurological complications in patients receiving V-A ECMO was high, which was closely related with adverse outcomes (including ICU LOS and 28-day mortality). Moreover, TNI, CRRT, and continuous NP > 12 h were independent risk indicators for neurological complications in V-A ECMO patients. And the neurological complications of patients during ECMO support had significant adverse effect on long-term surviving and neurological outcomes of patients after discharge for 6 months. Future work should include strict randomized clinical trials or substitutive research studies and stratification analyses to increase the understanding of the long-term neural prognosis and cognitive function of V-A ECMO patients.

## Data Availability Statement

The raw data supporting the conclusions of this article will be made available by the authors, without undue reservation.

## Ethics Statement

The studies involving human participants were reviewed and approved by Ethics Committee of Affiliated Hangzhou First People's Hospital, Zhejiang University School of Medicine. The ethics committee waived the requirement of written informed consent for participation.

## Author Contributions

All corresponding and first authors contributed to study concept and design. YnL, QG, XW, YwL, and WP: acquisition and analysis of data. YnL: writing of the original manuscript and statistical analysis. WH and SX: revision and editing of the manuscript. YZ and WH: material, technical, and administrative support, and supervision. All authors approved the final version of the manuscript and agree to be responsible for all aspects of the work.

## Conflict of Interest

The authors declare that the research was conducted in the absence of any commercial or financial relationships that could be construed as a potential conflict of interest.
