# Supplementary material for: Neurological Complications of Veno-Arterial Extracorporeal Membrane Oxygenation: A Retrospective Case-Control Study
Source: Front Med (Lausanne). 2021 Jul 1;8:698242. doi: 10.3389/fmed.2021.698242 (PMC8280317; doi:10.3389/fmed.2021.698242)
Supplement: Supplementary file 1 [file Table_1.docx]

**Supplementary Table S1. Comparison of** **post-ECMO related characteristics**

|  | **All patients** | **Neurological complications** | |  |
| --- | --- | --- | --- | --- |
|  | **n = 60** | **Yes (n = 39)** | **No (n = 21)** | **P** |
| **MAP (mmHg)** | | | | |
| 0h post-ECMO | 88.3 (73, 99) | 86.2 (73.8, 99.4) | 93 (72.5, 98.5) | 0.813 |
| 6h post-ECMO | 84.5 (75.2, 91.4) | 86 (74, 90.7) | 82.3 (75.3, 92.5) | 0.97 |
| 12h post-ECMO | 81.3 (73.3, 89.9) | 79.3 (71, 89.3) | 84.3 (79.3, 92.3) | 0.042 |
| 24h post-ECMO | 79 (72.1, 87.9) | 77.7 (71.7, 85.3) | 82.3 (73.8, 91.5) | 0.059 |
| 48h post-ECMO | 79.7 (73.3, 86.3) | 79.7 (73.3, 89.7) | 82 (72.8, 85.2) | 0.986 |
| 72h post-ECMO | 81.8 (72.1, 90) | 79 (70.7, 88.7) | 85.3 (75, 95.7) | 0.085 |
| **CVP (cmH_2_O)** | | | | |
| 0h post-ECMO | 10 (7,14) | 9 (7, 15) | 10.5 (6.75, 12.25) | 0.494 |
| 6h post-ECMO | 9 (6, 13.75) | 9.5 (6, 15) | 8.5 (6, 11.75) | 0.099 |
| 12h post-ECMO | 8 (6, 12.5) | 9 (6, 15.5) | 7 (5, 12) | 0.278 |
| 24h post-ECMO | 9 (6, 12) | 9.5 (6, 13) | 7 (5, 12) | 0.371 |
| 48h post-ECMO | 8.5 (6, 12) | 10 (6, 13) | 7 (5.5, 10) | 0.097 |
| 72h post-ECMO | 8 (5, 11.5) | 8 (5, 12) | 6.5 (4.25, 9) | 0.120 |
| **ScvO2 (mmHg)** | | | | |
| 0h post-ECMO | 74 (56.3, 86) | 71 (57.5, 84.2) | 77.2 (55.9, 88.3) | 0.678 |
| 6h post-ECMO | 75.2 (62.7, 84.7) | 73 (61.5, 84.7) | 76.4 (71.8, 86.5) | 0.163 |
| 12h post-ECMO | 77.1 (63.9, 83.9) | 71.3 (60, 81) | 82 (72.9, 84.4) | 0.021 |
| 24h post-ECMO | 74.5 (66.9, 82.9) | 70.9 (66.4, 80.3) | 81 (71, 84.7) | 0.091 |
| 48h post-ECMO | 75.1 (65.3, 81.1) | 74.7 (63.3, 85.5) | 76 (65.4, 78.7) | 0.986 |
| 72h post-ECMO | 68.3 (61.6, 73.4) | 68.3 (61.6, 73.4) | 67.9 (59, 75.8) | 1.000 |
| **Hypoxemia, n (%)** | | | | |
| 0h post-ECMO | 2 (3.3) | 2 (5.1) | 0 (0.0) | 0.537 |
| 6h post-ECMO | 1 (1.7) | 1 (2.6) | 0 (0.0) | 1.000 |
| 12h post-ECMO | 1 (1.7) | 1 (2.6) | 0 (0.0) | 1.000 |
| 24h post-ECMO | 0 (0.0) | 0 (0.0) | 0 (0.0) | / |
| 48h post-ECMO | 0 (0.0) | 0 (0.0) | 0 (0.0) | / |
| 72h post-ECMO | 0 (0.0) | 0 (0.0) | 0 (0.0) | / |
| **Hypercapnia, n (%)** | | | | |
| 0h post-ECMO | 3 (5.0) | 2 (5.1) | 1 (4.8) | 1.000 |
| 6h post-ECMO | 1 (1.7) | 1 (2.6) | 0 (0.0) | 1.000 |
| 12h post-ECMO | 1 (1.7) | 1 (2.6) | 0 (0.0) | 1.000 |
| 24h post-ECMO | 0 (0.0) | 0 (0.0) | 0 (0.0) | / |
| 48h post-ECMO | 0 (0.0) | 0 (0.0) | 0 (0.0) | / |
| 72h post-ECMO | 0 (0.0) | 0 (0.0) | 0 (0.0) | / |

Abbreviations: ECMO: extracorporeal membrane oxygenation; MAP: mean arterial pressure; CVP: Central Venous Pressure; ScvO2: Central venous oxygen saturation.

**Supplementary Table S2. Comparison of** **pre-ECMO baseline characteristics of patients**

|  | **All patients** | **Neurological complications** | |  |
| --- | --- | --- | --- | --- |
|  | **n=60** | **Yes (n=39)** | **No (n=21)** | **P** |
| **MAP (mmHg)** | 81 (69.3, 93.9) | 79.3 (66.1, 94.3) | 82.7 (69.8, 91.8) | 0.755 |
| **CVP (cmH_2_O)** | 11 (8, 16) | 11 (8, 16.5) | 11 (8.75, 13.75) | 0.579 |
| **PH** | 7.33 (7.22, 7.41) | 7.31 (7.21, 7.41) | 7.37 (7.32, 7.43) | 0.023 |
| **PaO2 mmHg)** | 108 (73, 210) | 99 (72, 192) | 179 (73, 477) | 0.243 |
| **PaCO2 (mmHg)** | 36.5 (28, 45) | 36.9 (28.6, 50.4) | 36.5 (26.8, 43.5) | 0.355 |
| **Hb (g/L)** | 142 (112, 156) | 142 (114.5, 157) | 142 (100, 156) | 0.463 |
| **PT (s)** | 14.2 (12.4, 17.6) | 14.2 (12.2, 17.2) | 14 (12.8, 19.8) | 0.704 |
| **APTT (s)** | 36.4 (30, 45.8) | 35.6 (29.4, 53.9) | 36.4 (31.3, 44.4) | 0.895 |
| **LAC (mmol/L)** | 3.2 (1.7, 7.3) | 3.9 (1.8, 9.7) | 2.1 (1.1, 4.7) | 0.144 |

Abbreviations: ECMO: extracorporeal membrane oxygenation; MAP: mean arterial pressure; CVP: central venous pressure; PH: Potential of Hydrogen; PaO_2:_ Arterial oxygen partial pressure; PaCO_2:_ Arterial carbon dioxide partial pressure. Hb: hemoglobin; PT: prothrombin time; APTT: activated partial thromboplastin time; LAC: lactic acid.

**Supplementary Table S3. Comparison of serum creatinine of patients**

| **Cr (μmol/L)** | **All patients** | **Neurological complications** | |  |
| --- | --- | --- | --- | --- |
|  | **n = 60** | **Yes (n = 39)** | **No (n = 21)** | **P** |
| Pre-ECMO | 102.6 (78.2, 140.7) | 110 (78.5, 156.5) | 94 (75, 113) | 0.603 |
| 0h post-ECMO | 108 (86.5, 147) | 110 (93.5, 179) | 93 (75.5, 118.2) | 0.112 |
| 6h post-ECMO | 101.5 (80.5, 125) | 105 (82, 140) | 94 (78.5, 115) | 0.055 |
| 12h post-ECMO | 109 (89, 147) | 124 (93.7 166) | 91 (78.5, 111) | 0.002 |
| 24h post-ECMO | 105.5 (84.5, 134) | 108 (94, 156) | 102 (75, 117.5) | 0.083 |
| 48h post-ECMO | 105.5 (85, 124.5) | 87 (108, 134) | 87 (70, 115) | 0.032 |
| 72h post-ECMO | 103 (78.5, 123.5) | 111 (93.5, 135) | 81.5 (68.7, 114) | 0.016 |

Abbreviations: ECMO: extracorporeal membrane oxygenation; Cr: creatinine.
